# The Human Electric Battery

**Published:** 1898-05

**Authors:** 


### THE HUMAN ELECTRIC BATTERY.

The superstition that human beings should sleep with their heads to the north is believed by the French to have for its foundation a scientific fact. They affirm that each human system is in itself an electric battery, the head being one of the electrodes, the feet the other. Their proof was discovered from experiments which the Academy of Sciences was allowed to make on the body of a man who was guillotined.

This was taken the instant it fell and placed upon a pivot free to move as it might. The head part, after a little vacillation, turned to the north, and the body then remained stationary. It was turned half way round by one of the professors, and again the head end of the trunk moved slowly to the cardinal point due north, the same results being repeated until the final arrestation of organic movement.—*Railroad Telegrapher.*

